# Antibody responses after sequential vaccination with PCV13 and PPSV23 in kidney transplant recipients

**DOI:** 10.1007/s15010-023-02054-3

**Published:** 2023-05-27

**Authors:** Nils Mülling, Lukas van de Sand, Kim Völk, Ulrich Wilhelm Aufderhorst, Mark van der Linden, Peter A. Horn, Andreas Kribben, Benjamin Wilde, Adalbert Krawczyk, Oliver Witzke, Monika Lindemann

**Affiliations:** 1 Department of Nephrology, University Hospital Essen, University Duisburg-Essen, Essen, Germany; 2 Department of Infectious Diseases, University Hospital Essen, University Duisburg-Essen, Essen, Germany; 3 Institute for Transfusion Medicine, University Hospital Essen, University Duisburg-Essen, Essen, Germany; 4 Department of Medical Microbiology, German National Reference Center for Streptococci, University Hospital Aachen (RWTH), Aachen, Germany

**Keywords:** *Streptococcus pneumoniae*, Vaccination, Kidney transplantation, Sequential vaccination, Serotype specific immunity, Pneumococcal antigens

## Abstract

**Purpose:**

Vaccination against *Streptococcus pneumoniae* is recommended in transplant recipients to reduce the morbidity and mortality from invasive pneumococcal disease. Previous studies indicate that transplant recipients can produce specific antibodies after vaccination with the 13-valent pneumococcal conjugate vaccine Prevenar 13 (PCV13) or the pneumococcal polysaccharide vaccine Pneumovax 23 (PPSV23). National guidelines recommend sequential vaccination with PCV13 followed by PPSV23 in kidney transplant patients. However, there are currently no data on the serological response in kidney transplant recipients, who received a sequential vaccination with PCV13 and PPSV23.

**Methods:**

In the current study, we sequentially vaccinated 46 kidney transplant recipients with PCV13 and PPSV23 and determined global and serotype-specific anti-pneumococcal antibody responses in the year following vaccination.

**Results:**

Serotype-specific and global anti-pneumococcal antibody concentrations were significantly higher compared to baseline. We observed that serotype-specific antibody responses varied by serotype (between 2.2- and 2.9-fold increase after 12 months). The strongest responses after 12 months were detected against the serotypes 9N (2.9-fold increase) and 14 (2.8-fold increase). Global antibody responses also varied with respect to immunoglobulin class. IgG2 revealed the highest increase (2.7-fold), IgM the lowest (1.7-fold). Sequential vaccination with both vaccines achieved higher antibody levels in comparison with a historical cohort studied at our institute, that was vaccinated with PCV13 alone. During the 12-months follow-up period, none of the patients developed pneumococcal-associated pneumonia or vaccination-related allograft rejection.

**Conclusion:**

In conclusion, we strongly recommend sequential vaccination over single immunization in kidney transplant recipients.

## Introduction

Immunocompromised patient cohorts such as solid organ transplant recipients are at major risk for infectious complications. These include lower respiratory tract infections, which can lead to severe disease with requirement of hospital treatment or even transfer to the intensive care unit [1]. *Streptococcus pneumoniae* (*S. pneumoniae*) is a capsulated gram-positive bacterium, that frequently colonizes the human nasopharynx, but can also lead to local and systemic diseases [2].

Apart from meningitis, otitis media and sinusitis, it is the most frequently identified bacterial pathogen in pneumonia [3]. Due to the administration of immunosuppressive agents, the risk of invasive pneumococcal disease (IPD) is dramatically increased in solid organ recipients. Therefore, vaccination in immunocompromised individuals is recommended to reduce the incidence of IPD [4–6].

The capsule is the main virulence factor of *S. pneumoniae* and consists of different polysaccharides, which form the basis for the classification of pneumococci into over 90 serotypes. Twenty-three of these serotypes are responsible for 80–90% of infections nowadays [7].

Currently, two types of pneumococcal vaccines are licensed and used in routine clinical practice: the pneumococcal polysaccharide vaccines (PPSV) and pneumococcal conjugate vaccines (PCV). PPSVs act as T-cell independent type 2 antigens, inducing IgG responses and poor generation of memory B cells. PCV was developed to enhance immunogenicity by covalent conjugation to carrier proteins. These peptides induce a T helper cell response, which can promote B-cell differentiation into antibody producing plasma cells or memory B cells [8].

Apart from a reduced immunogenicity of vaccines in solid organ transplant recipients, another concern refers to the risk of triggering allograft rejection through stimulation of alloreactive T and B cells, which is of particular interest in PCVs as they are specifically engineered to increase immune activation [8, 9].

In Germany, a sequential administration of the 13-valent pneumococcal conjugate vaccine (PCV13) followed by the 23-valent pneumococcal polysaccharide vaccine (PPSV23) after 6–12 months is recommended for risk groups including solid organ recipients [6]. It is recommended to control humoral vaccination responses in this cohort. However, it is still unclear to what extent serological titers reflect protection against an infection with *S. pneumoniae* [6]. Studies in kidney transplant recipients (KTR), that examined the humoral response after administration of PCV13 revealed increased functional antibody responses after vaccination [8, 10] but could not match the responses in healthy controls [11]. The administration of PPSV23 in kidney KTR also led to a significant increase of antibodies, which was still detectable after a period of 15 months [12, 13].

However, there are currently no data on the serological response in KTR, who received a sequential vaccination with PCV13 and PPSV23. In addition to a measurement of a global pneumococcal antibody response (against 23 serotypes), we also determined specific immune responses to six pneumococcal serotypes. Therefore, this study aims to investigate the serological immunogenicity and safety of the aforementioned vaccination regiment in KTR.

## Materials and methods

### Study population

A total of 46 kidney transplant recipients were included in this single-center study between 11/2018 and 10/2019. Basic patient information is given in Table 1. Median age was 57 years (range 22–76 years). Thirty-one patients were male, fifteen patients were female. The median interval between the last kidney transplantation and study inclusion was 38 months (range 3–338 months).

**Table 1:** Basic patient characteristics

Parameter	Median (Range) or Number (No.)
Median age (range), years^a^	57 (22–76)
Patient sex (male/female)	31/15
Median interval after kidney transplantation (range), months	38 (3–338)
Median serum creatinine (range), mg/dl	
Pre vaccination	1.58 (0.87–3.55)
Month 6 post vacc	1.55 (0.56–3.65)
Month 12 post vacc	1.57 (0.86–3.87)
Immunosuppression, no. (%)^a^	
Cyclosporine A	6 (13%)
Tacrolimus	35 (76%)
Mycophenolic acid	33 (72%)
mTOR inhibitors	7 (15%)
Corticosteroids	41 (89%)
Belatacept	3 (7%)
Kidney transplantation, no. (%)	
First	42 (91%)
Second	4 (9%)
Comorbidities, no. (%)	
Diabetes mellitus	11 (24%)
Hypertension	31 (67%)
Coronary heart disease	10 (22%)
Co-medication, no. (%)^a^	
Diuretics	18 (39%)
ACE inhibitors/AT1 receptor antagonists	24 (52%)
Calcium chanel blockers	24 (52%)
Beta blockers	29 (63%)
Statins	28 (61%)
Oral anticoagulation	5 (11%)
Insulin	7 (17%)

^a^At the time of first blood sampling; mTOR = mammalian target of rapamycin

The following criteria led to exclusion from the study:

- Interval between kidney transplantation and study inclusion < 3 months
- eGFR < 15 ml/min/1.73 m^2^
- Acute deterioration of allograft function (related to the definition of acute kidney injury) [14]:
  ○ Increase in serum creatinine by ≥ 0.3 mg/dl within 48 h or
  ○ Increase in serum creatinine to 1.5 times baseline within the prior 7 days or
  ○ Urine volume < 0.5 ml/kg/h for 6 h
- Acute symptomatic bacterial infection with fever > 38.5 degrees
- Allograft rejection within last 6 months
- Pregnancy
- Previous vaccination against *S. pneumoniae* within the last five years

**Fig. 1:**
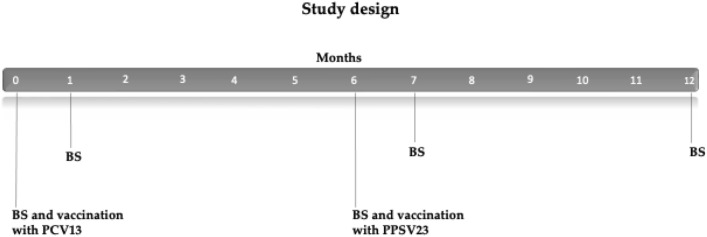
Study design. All patients sequentially received a single dose of PCV13 and PPSV23. Blood samples (BS) were drawn at the indicated time points

The study design is summarized in Fig. 1. Patients were sequentially vaccinated with a single dose of PCV13 and a single dose of PPSV23 six months later. Blood samples were drawn immediately prior to both vaccinations and at months 1, 7 and 12 post-vaccination, respectively. The patients were followed up for clinical endpoints such as pneumonia or allograft rejection until month 18 after the first vaccination.

This study was approved by the institutional review board of the University Hospital Essen (14–5858-BO), and written informed consent was obtained from all participants. It was carried out in accordance with the Declarations of Helsinki and Istanbul and its subsequent amendments.

### Vaccines

The pneumococcal vaccine Prevenar 13 contains polysaccharides of 13 serotypes (1, 3, 4, 5, 6A, 6B, 7F, 9 V, 14, 18C, 19F, 19A, and 23F), each conjugated to a nontoxic mutant form of diphtheria toxin cross-reactive material 197 (CRM197). It contains 2.2 μg/dose of each of the serotypes, except for serotype 6B at 4.4 μg/dose (0.5 mL) [10]*.*

The pneumococcal vaccine Pneumovax 23 is an unconjugated vaccine that contains 25 µg each of the 23 pneumococcal serotypes 1, 2, 3, 4, 5, 6B, 7F, 8, 9N, 9 V, 10A, 11A, 12F, 14, 15B, 17F, 18C, 19F, 19A, 20, 22F, 23F, and 33F. Further details on the vaccines have been described in detail previously [15]. Both vaccines were injected into the deltoid muscle [10]*.*

### Control groups

We considered two historical control groups, tested for pneumococcal antibodies by the same commercial ELISA as the current study population (VaccZyme™, The Binding Site, Schwetzingen, Germany). The first cohort comprises 47 clinically stable adult kidney transplant recipients, tested at our transplant center after a single vaccination with PCV13 [11, 16]. The second cohort comprises vaccine naïve healthy blood donors described in two previous studies, where reference values were defined for IgG and IgG2 [17] or for IgA and IgM [18]. A third historical control group, healthy controls that received a single vaccination with PCV13 [19], was also considered to compare pneumococcal serotype-specific IgG antibodies.

### Measurement of serotype-specific antibody concentrations

Pneumococcal serotype-specific IgG antibody levels were determined for 6 serotypes (2, 3, 6A, 9N, 11A, 14) by a serotype-specific enzyme-linked immunosorbent assay (ELISA). Isotype-specific reference ELISA was performed according to the WHO protocol published at https://www.vaccine.uab.edu/uploads/mdocs/ELISAProtocol(007sp).pdf. In summary, 96-well microtiter plates (Greiner Bio-One, Frickenhausen, Germany) were coated with serotype specific pneumococcal polysaccharide (Pn PS) antigen (ATCC, Manassas, VA, USA) and incubated in a humidified chamber at 37 °C for 5 h. Additionally, plates were blocked for 1 h at room temperature with DPBS plus 1% (*w*/*v*) dried nonfat milk powder prior to use. Patient serum samples and standard reference serum (007sp) were pre-incubated with cell-wall polysaccharide (CWPS) and pneumococcal type 22F capsular polysaccharide. Quality control serum was kindly provided by Mustafa Akkoyunlu (Pneumococcal Reference Laboratory, Birmingham, AL, USA). Control and test sera were added to the plate in duplicate wells and incubated at room temperature. The antibodies bound to the plates were detected by an anti-Human IgG (γ-chain specific) F(ab')2-Fragment-Peroxidase for all 4 IgG subclasses (A2290; Sigma-Aldrich, St. Louis, MO, USA). Subsequently TMB ELISA substrate (Thermo Fisher Scientific, Cleveland, OH, USA) reaction was stopped by addition of a sulphonic acid stop solution. Optical density was measured at 450 nm and 690 nm (reference) using Tristar 3 multimode ELISA plate reader (Berthold Technologies, Bad Wildbad, Germany). Serum antibody concentrations were calculated with GraphPad Prism 8.4.2.679 (GraphPad Prism Software, San Diego, CA, USA) by a log linear regression analysis. The lower limit of detection of this assay is approximately 0.01 mg/L.

### Determination of global antibody responses against pneumococci

Antibodies against *S. pneumoniae* were determined by four commercial ELISA formats which detect IgG, IgG2, IgA and IgM antibodies against 23 pneumococcal serotypes (VaccZyme™), which are the same serotypes as those included in the PPSV23. This assay, which we called global pneumococcal ELISA, was performed according to the manufacturer’s instructions.

### Statistical analysis

Data were analyzed using GraphPad Prism or IBM SPSS Statistics version 25 (Armonk, NY, USA). We first checked the data for normal distribution using Shapiro–Wilk test. As various variables did not reveal normal distribution, we applied non-parametric methods to further analyze our data. ELISA responses at different time points were analyzed using the Kruskal–Wallis test, with Dunn´s multiple comparisons test. Continuous variables were compared using the Mann–Whitney *U* test. Correlation analyses of numerical variables were performed using the Spearman test (two-tailed). The impact of MPA treatment and the interval between transplantation and first vaccination on pneumococcal antibodies was analyzed using multivariate analysis (logistic regression). If not otherwise stated, median values are indicated. Results were considered significant at *p* < 0.05.

## Results

### Clinical course of the study population

All patients were clinically examined until month 18 after the first vaccination. No hospitalized pneumonia with detection of *S. pneumoniae* occurred. One patient had a bacterial superinfection after pneumonia caused by influenza virus, but no certain bacterial pathogen could be identified. Donor-specific antibodies (DSA) were analyzed in 43 patients during the study period; one patient developed de novo DSA 1.5 months after first vaccination (MFI 1600), but no allograft rejection was detected in this patient. A biopsy proved rejection was detected in another patient (histopathological: borderline rejection (Banff category 3), who did not develop de novo DSA.

### Pneumococcal antibodies prior to and post vaccination

**Fig. 2:**
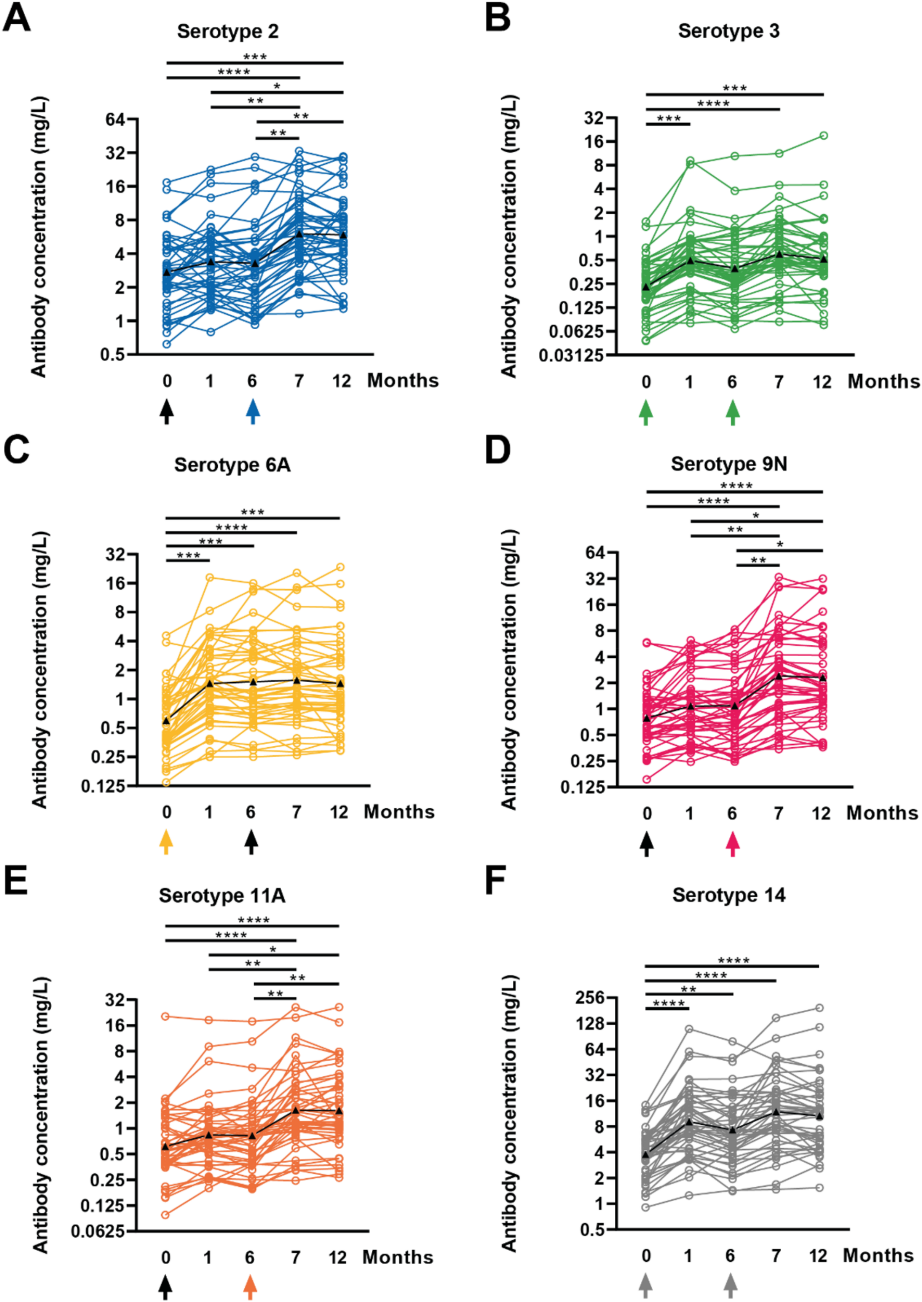
Individual time courses of serotype-specific pneumococcal antibodies prior to vaccination and after vaccination with PCV13 (at month 0) and with PPSV23 (at month 6). Panels (**A–F**) show serotypes 2, 3, 6A, 9N, 11A and 14. The time of vaccination is indicated by an arrow. Colored arrows denote that the vaccine contains the corresponding serotype. The black line with triangles indicates the geometric mean values. The results are shown on a logarithmic scale (log2). Data in 46 kidney transplant recipients were analyzed by Kruskal–Wallis test, with Dunn´s multiple comparisons test. *p < 0.05, **p < 0.01, ***p < 0.0005, ****p < 0.0001

**Fig. 3:**
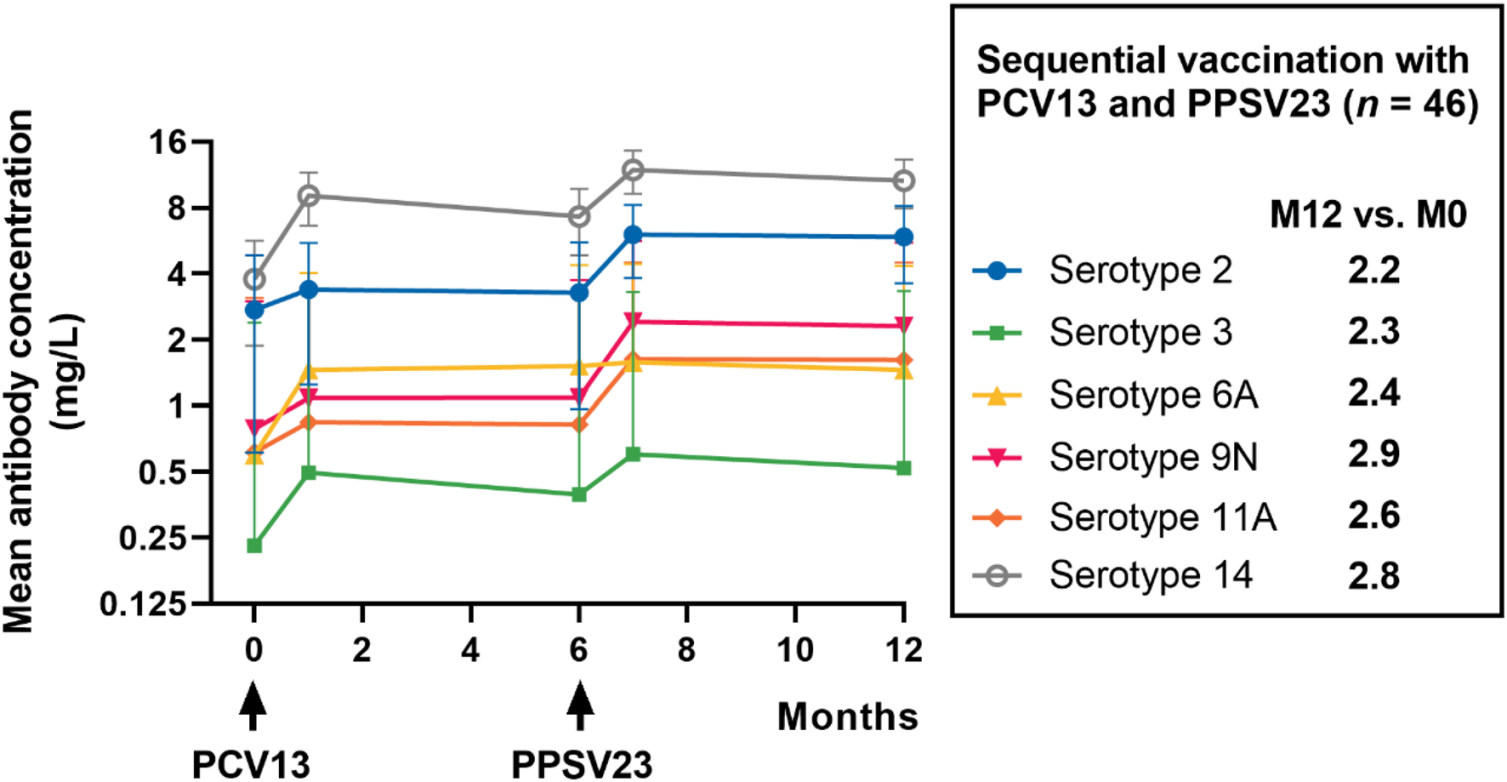
Concentrations of serotype-specific pneumococcal antibodies in time course of 12 months after first vaccination. Antibodies were determined prior to first vaccination with PCV13 (M0), 1 month after (M1), prior to second vaccination with PPSV23 (M6), at month 7 (M7) and month 12 (M12) thereafter for 46 kidney transplant recipients. Pneumococcal antibodies are given as geometric mean concentration and geometric standard deviation factor. The time of vaccination is indicated by an arrow

We tested 230 sera from 46 patients by the standardized WHO pneumococcal ELISA (Fig. 2). Serotype-specific antibody concentrations were determined for a total of six serotypes present in either PCV13 (serotype 6A), PPSV23 (serotypes 2, 9N, 11A), or both vaccines (serotypes 3, 14). Comparing pre-vaccination and month 12 antibody concentrations, a significant increase was observed for each serotype (*p* < 0.0005). Highest values were measured generally at month 7, 1 month after vaccination with PPSV23. In the course of 6 months, there was no significant (n.s.) decrease of antibody levels after either of the two vaccinations. Geometric mean concentration (GMC) was lowest and increased second lowest for serotype 3. The GMC of serotype 3 antibodies increased from 0.2 mg/L pre-vaccination to 0.5 mg/L at month 1 (*p* < 0.0005) and showed highest values with 0.6 mg/L at month 7 (*p* < 0.0001). Only serotype 2 antibodies showed a smaller percentage increase. At pre-vaccination, the GMC of serotype 2 antibodies was 2.7 mg/L which raised up to 6.0 mg/L at month 7 (*p* < 0.0001). As expected, no significant increase was observed in the first 6 months after vaccination with PCV13 for antibody levels against serotypes 2, 9N and 11A, as these are only present in PPSV23. However, serotype 9N showed the strongest increase (2.9-fold) compared to the initial value (Figs. 2, 3). At month 7 after study initiation (1 month after administration of the second vaccination), antibody concentrations against serotype 9N were constant at 2.3 mg/L (*p* < 0.0001). Serotype 14, which has been vaccinated twice as this serotype is included in both PCV13 and PPSV23, reached the highest antibody concentrations. At month 7 it reached a GMC of 11.9 mg/L (*p* < 0.0001). The 2.8-fold increase of antibodies against serotype 14 was the second strongest. A less than twofold increase in concentrations of antibodies against all six serotypes compared to baseline, was observed in only 3 out of all 46 patients. Detailed time courses of antibody concentrations before and after vaccination are presented in Table 2. For the six serotypes, the twofold increase rate of anti-pneumococcal antibodies varied from 54.4% to 71.7% at month 12 (Table 2).

**Table 2:** Serologic responses to sequential pneumococcal vaccination

Serotype	2	3	6A	9N	11A	14
Geometric mean concentration (IQR) mg/L, *n* = 46 Geometric mean fold increases (IQR), *n* = 46						
Month 0	2.7 (1.9–4.3)	0.2 (0.1–0.3)	0.6 (0.3–0.9)	0.7 (0.4–1.2)	0.6 (0.3–1.0)	3.7 (2.3–5.5)
Month 1	3.3 (1.9–5.2)	0.5 (0.3–0.8)	1.4 (0.7–2.8)	1.0 (0.5–1.7)	0.8 (0.5–1.1)	9.0 (4.4–15.8)
	1.2 (0.8–1.5)	2.1 (1.6–2.6)	2.4 (1.7–3.3)	1.3 (0.9–1.7)	1.3 (0.9–2.0)	2.4 (1.5–3.0)
Month 6	3.2 (1.7–5.1)	0.3 (0.2–0.7)	1.5 (0.7–3.1)	1.0 (0.5–1.9)	0.8 (0.4–1.2)	7.3 (4.4–12.4)
	1.2 (0.8–1.6)	1.7 (1.0–2.3)	2.5 (1.6–3.6)	1.3 (0.9–1.9)	1.3 (0.9–2.1)	1.9 (1.1–2.9)
Month 7	6.0 (3.5–9.9)	0.6 (0.3–1.1)	1.5 (0.8–3.0)	2.4 (1.0–6.2)	1.6 (0.9–2.8)	11.8 (6.6–20.0)
	2.2 (1.4–8.2)	2.6 (1.7–3.9)	2.6 (1.7–3.8)	3.0 (1.7–5.3)	2.6 (1.6–3.9)	3.1 (1.9–4.4)
Month 12	5.8 (3.9–8.6)	0.5 (0.3–0.9)	1.4 (0.7–2.7)	2.3 (1.1–5.5)	1.6 (0.8–3.2)	10.6 (5.1–18.7)
	2.1 (1.4–9.3)	2.2 (1.3–3.8)	2.4 (1.6–3.6)	2.9 (1.6–4.1)	2.6 (1.3–4.3)	2.8 (1.8–3.8)

**Fig. 4:**
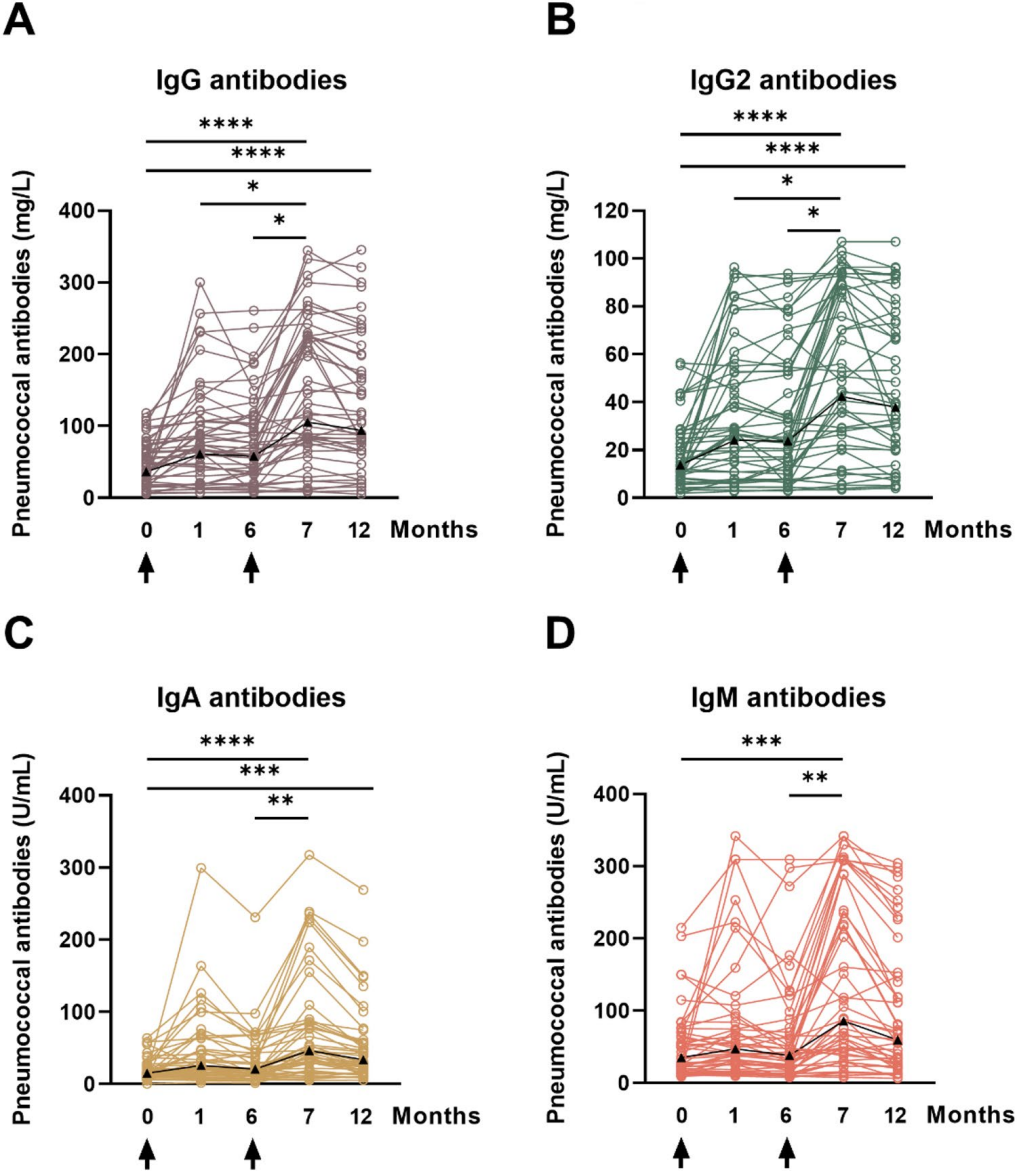
Individual time courses of pneumococcal antibodies prior to vaccination and after vaccination with PCV13 (at month 0) and with PPSV23 (at month 6). The time of vaccination is indicated by an arrow. Panel (**A**) shows IgG antibodies, panel (**B**) IgG2, panel (**C**) IgA and panel **(D)** IgM, as determined by a commercially available ELISA, measuring antibodies against 23 serotypes (global ELISA). Data in 46 kidney transplant recipients were analyzed by Kruskal–Wallis test, with Dunn´s multiple comparisons test. *p < 0.05, **p < 0.01, ***p < 0.0005, ****p < 0.0001

**Fig. 5:**
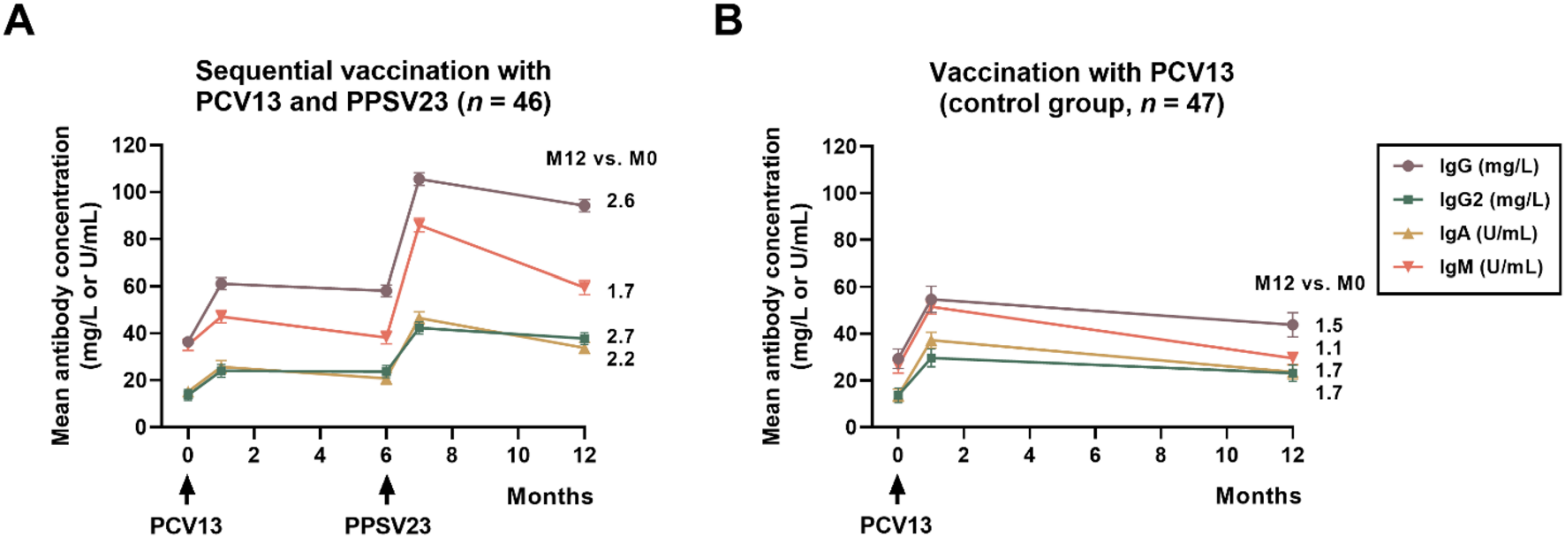
Time courses of pneumococcal antibodies in kidney transplant recipients. Panel (**A**) shows data of the current cohort of 46 patients vaccinated sequentially with PCV13 (at month 0) and with PPSV23 (at month 6). Panel (**B**) displays the course of antibodies in a historical control group of 47 kidney transplant recipients from our transplant center after a single vaccination with PCV13 (at month 0) [11, 16]. Both cohorts were tested for IgG, IgG2, IgA and IgM antibodies by the same commercially available ELISA, measuring antibodies against 23 serotypes (global ELISA). Pneumococcal antibodies are given as geometric mean concentration and geometric standard deviation factor. The time of vaccination is indicated by an arrow

The global IgG, IgG2, IgM and IgA ELISA were performed for 23 serotypes in 230 serum samples. The GMC of IgG antibodies against 23 serotypes was 36.5 mg/L pre-vaccination, 61.1 mg/L at month 1, 58.1 mg/L at month 6, 105.5 mg/L at month 7 and 94.3 mg/L at month 12 (Figs. 4A, 5A, Table 3). We observed a significant increase (*p* < 0.0001) of IgG antibodies at month 7 and 12, as compared to baseline. Moreover, responses at month 1 and 7 and at month 6 and 7 differed significantly (*p* < 0.05), i.e., antibody levels increased after receiving the second vaccination (with PPSV23). The course of IgG2 and IgG antibody levels was almost identical, however at lower concentrations, as expected (Figs. 4A, B, 5A). IgA and IgM antibody levels also showed a significant increase at month 7, after two vaccinations (Figs. 4C, 4D, 5A). Whereas IgA antibody levels remained significantly increased at month 12, as compared to baseline, the increase of IgM antibody levels was no longer significant at month 12.

**Table 3:** Time course of pneumococcal antibody levels in 46 kidney transplant recipients vaccinated with PCV13 at month 0 and with PPSV23 at month 6 and comparison of their antibody concentrations with a healthy reference cohort

Antibody subclass	IgG		IgG2		IgA		IgM	
Time point	GMC	PRC	GMC	PRC	GMC	PRC	GMC	PRC
Month 0	36.5	46	13.8	34	15.2	29	35.1	34
Month 1	61.1	71	24.1	63	25.7	51	47.1	46
Month 6	58.1	66	23.7	61	20.8	49	38.2	34
Month 7	105.5	83	42.3	80	46.5	76	86.0	71
Month 12	94.3	83	37.9	80	33.9	68	59.5	61

*GMC* geometric mean concentration (given for IgG and IgG2 as mg/L and for IgA and IgM as U/mL), *PRC* percentage comparable to reference cohort

The reference group comprises vaccine naïve healthy blood donors. Their pneumococcal antibodies were used for comparison. Reference values were defined as ≥ 43.8 mg/L for IgG [17], ≥ 20.5 mg/L for IgG2 [17], ≥ 21.0 U/ml for IgA [18] and ≥ 54.0 U/ml for IgM [18]), respectively.

The course of antibodies was compared with an independent control group of kidney transplant recipients, tested at our transplant center after a single vaccination with PCV13 between 2014 and 2015 [11] (Fig. 5). This cohort had a median age of 53 years and the median interval between kidney transplantation and vaccination was 49 months. Data after vaccination with PCV13 were overall very similar in the current cohort and the historical control group. Follow-up data at month 12 indicate that after sequential vaccination geometric mean concentrations of IgG, IgG2, IgA and IgM antibodies were 2.6-fold, 2.7-fold, 2.2-fold, and 1.7-fold higher than prior to vaccination, respectively (Fig. 5A). After a single vaccination with PCV13, we observed 1.5-, 1.7-, 1.7-, and 1.1-fold higher antibodies at month 12 as compared to baseline, respectively (Fig. 5B).

Next, we compared antibody concentrations of our patient cohort with two historical groups of healthy controls. To evaluate global anti-pneumococcal antibody concentrations, we analyzed which fraction of patients showed antibody concentrations comparable to a healthy, vaccine naïve reference group (GMC defined as ≥ 43.8 mg/L for IgG [17], ≥ 20.5 mg/L for IgG2 [17], ≥ 21.0 U/ml for IgA [18] and ≥ 54.0 U/ml for IgM [18]). Prior to vaccination, 29–46% of the patients exceeded the respective threshold, depending on the subclass of pneumococcal antibodies (Table 3). At month 7, when the maximum response was achieved, 83% of patients had IgG antibody levels comparable to those of the reference group, and 80% had comparable IgG2, 76% IgA and 71% IgM antibody concentrations. In summary, kidney transplant recipients display lower baseline-levels of global anti-pneumococcal antibody concentrations across all subclasses compared to healthy controls. However, after sequential vaccination, the majority of kidney transplant recipients show higher antibody concentrations than vaccine naïve healthy controls. The serotype-specific antibody concentrations were compared to another group of healthy individuals who received a single dose of PCV13 [19]. With respect to the tested serotypes, we could compare the values of serotypes 3, 6A and 14. One month after vaccination with PCV13, healthy individuals displayed higher antibody concentrations than our patient cohort (serotype 3: 1.4 vs. 0.5 mg/L; serotype 6A: 7.9 vs. 1.4 mg/L; serotype 14: 12.0 vs. 9.0 mg/L). Twelve months after receiving PCV13, GMC of serotype 6A (only part of PCV13) was still higher in healthy individuals (4.4 mg/L vs. 1.4 mg/L), whereas antibody concentrations of serotypes 3 and 14 (part of PCV13 and PPSV23) were similar or even lower compared to our study population (serotype 3: 0.6 mg/L vs. 0.5 mg/L; serotype 14: 7.7 mg/L vs. 10.6 mg/L).

**Fig. 6:**
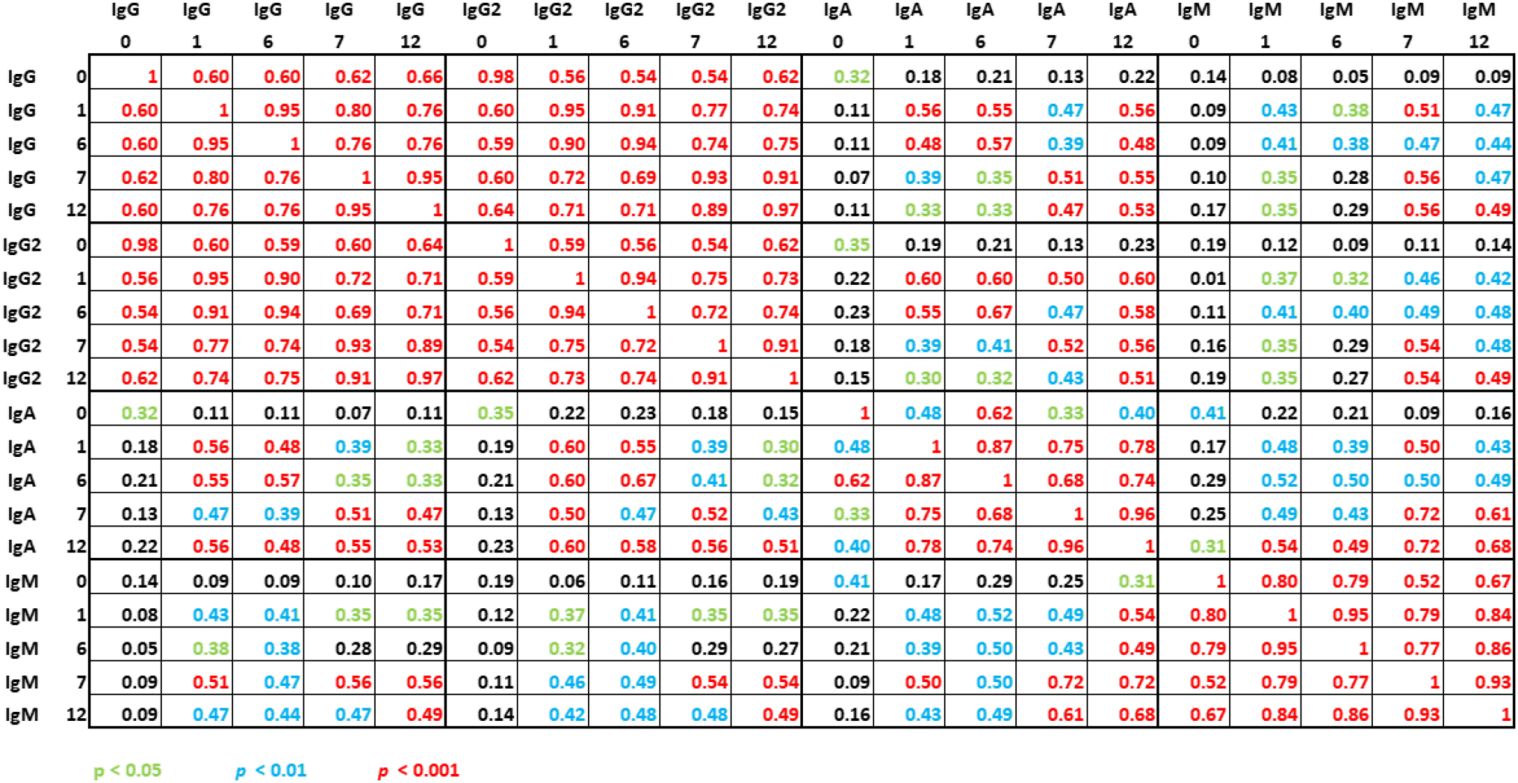
Spearman correlation analysis of pneumococcal antibodies in 46 kidney transplant recipients vaccinated sequentially with PCV13 (at month 0) and with PPSV23 (at month 6). IgG, IgG2, IgA and IgM antibody concentrations were determined using a commercially available ELISA, measuring antibodies against 23 serotypes (global ELISA). The time points (month 0-month 12) are indicated by numbers (0–12) in the heading. The numbers in the table indicate correlation coefficients, which are color-coded according to the level of significance

Spearman correlation analysis of pneumococcal antibody concentrations at various time points and of various subclasses showed the strongest correlation between IgG and IgG2 antibody concentrations, reaching statistical significance (*p* < 0.001) at all time points as indicated by red color (Fig. 6). Overall, IgG and IgG2 antibody levels showed stronger correlation with IgA than with IgM antibody levels. Within each subclass, antibody levels at month 0 correlated significantly with all subsequent time points. Thus, pneumococcal antibody levels prior to vaccination were predictive of antibody levels after vaccination, reaching the highest correlation coefficients for IgM antibody levels.

We also performed correlation analyses between global IgG values and serotype specific antibody concentrations at the respective time-points. With exception of serotype 2 a significant correlation was detectable at most time points after vaccination, reaching the highest correlation coefficients for serotype 9N (Table 4).

**Table 4:** Spearman correlation analysis of global IgG values and serotype specific IgG concentrations

	IgG 0	IgG 1	IgG 6	IgG 7	IgG 12
Pn PS-2	0.18	0.19	0.16	0.19	0.23
Pn PS-3	0.23	0.39	0.42	0.39	0.18
Pn PS-6A	0.10	0.37	0.39	0.32	0.30
Pn PS-9N	0.31	0.39	0.39	0.47	0.46
Pn PS-11A	0.19	0.27	0.32	0.37	0.33
Pn PS-14	0.19	0.40	0.40	0.29	0.32

Each column shows the correlation coefficient for global IgG values and serotype specific IgG concentrations at the same time point (month 0–12, indicated as 0–12)

*p* < 0.05, *p* < 0.01

### Association between clinical patient characteristics and pneumococcal antibody levels

Global IgG, IgG2, IgM and IgA antibody concentrations did not differ significantly between male and female patients at any time-point. There was also no significant correlation between age or allograft function (creatinine, eGFR) and global antibody concentrations. However, Spearman analysis indicated a significant positive correlation between global IgG antibody levels and interval between (last) transplantation and first vaccination or subsequent follow-up analyses, with the highest correlation coefficient at month 12 (*r* = 0.48, *p* = 0.01). We also observed a positive correlation between this interval and global IgG2 antibody levels reaching statistical significance at month 7 (*r* = 0.40, *p* = 0.006) and month 12 (*r* = 0.42, *p* = 0.003). Thus, patients vaccinated later after transplantation had higher anti-pneumococcal IgG and IgG2 responses, especially in the long-term follow-up at month 12. However, this observation did not reach statistical significance in the cases of global IgA and IgM antibody levels.

**Fig. 7:**
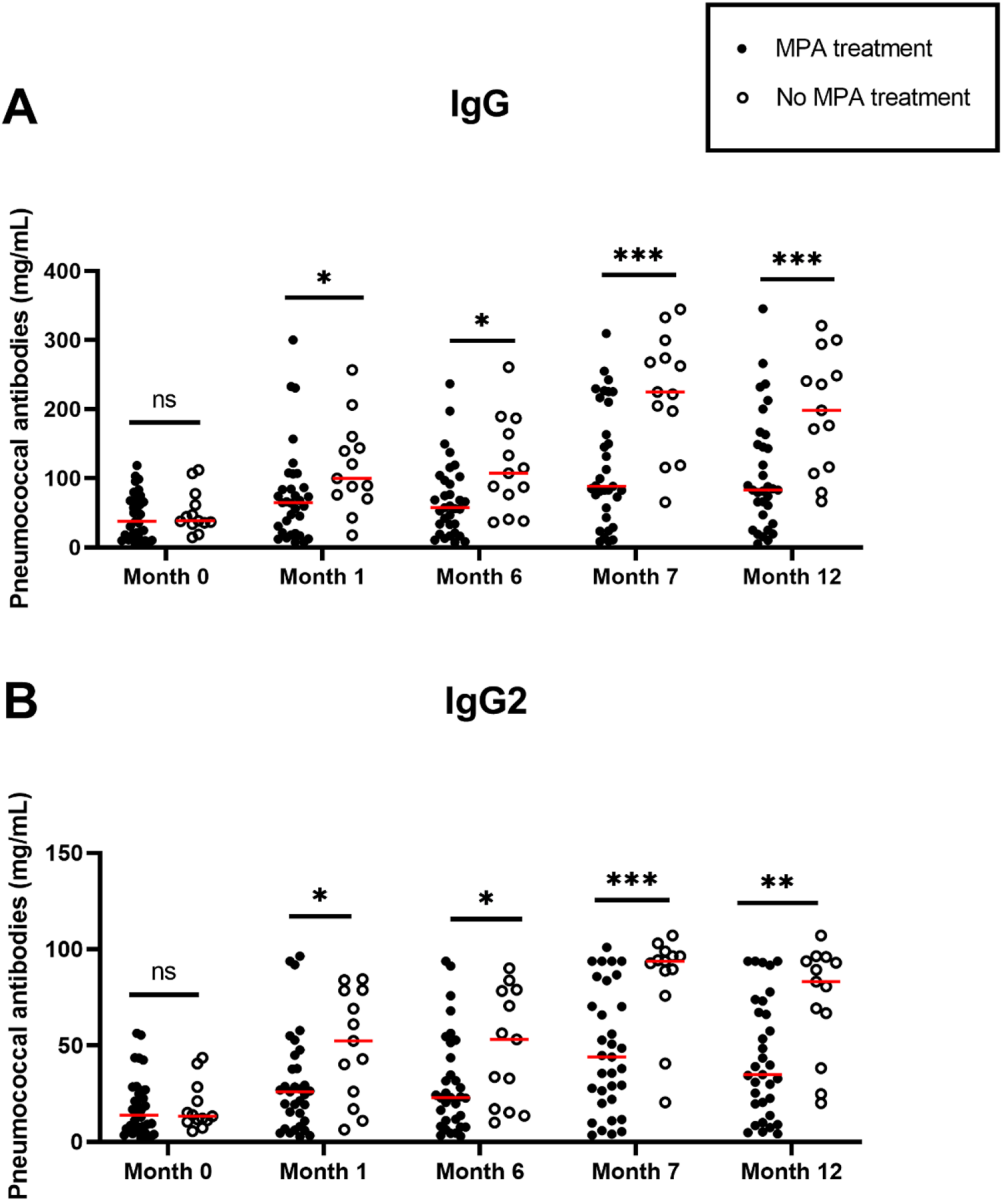
Comparison of global IgG (**A**) and IgG2 (**B**) antibody responses between patients with (n = 33) and without mycophenolic acid (MPA) treatment (n = 13). Mann–Whitney U test was performed for comparison between groups. After logistic regression analysis, only differences at month 7 and month 12 proved to be significant for both antibody classes. Red lines indicate median values. *p < 0.05, **p < 0.01, ***p < 0.0005

We also analyzed the association between the immunosuppressive regiment and global antibody responses. As the vast majority of patients had a calcineurin inhibitor-based scheme (Table 1), we could not perform an analysis for this treatment, but we compared patients with and without intake of mycophenolic acid (MPA). Patients who did not take MPA had significantly higher global IgG and IgG2 antibody levels at all time-points after vaccination (Fig. 7). There was no significant difference between both groups regarding global IgM and IgA antibody levels. As only the interval between transplantation and vaccination and MPA treatment revealed significant differences in the current study, we included both as independent variables in a logistic regression model to further determine their impact on global IgG and IgG2 antibody levels. The analysis demonstrated that only treatment of MPA proved a significant association with global IgG and IgG2 antibody levels at months 7 and 12.

## Discussion

The current study examined the serological immunogenicity and safety of a sequential vaccination with PCV13 and PPSV23 in kidney transplant recipients. We could demonstrate that this vaccination regiment induced a serological response indicated by a significant increase in both, global and serotype specific antibody levels. We recorded only one event each of biopsy proven rejection and de novo development of DSA in our cohort during a follow-up of 18 months after the first vaccination (with PCV13). Given the general risk of alloimmune processes in solid organ transplant recipients, we do not ascribe these events to the administered vaccines [20]. Therefore, we evaluate the sequential administration of both vaccines as immunologically safe in our cohort.

The age of patients in this study ranged from 22 to 76 years, with a median age of 57 years. For patients under and over 65 years of age, the Centers for Disease Control and Prevention (CDC) recommends different vaccination intervals. For younger patients, vaccination intervals vary depending on their specific risk factor, but should not be less than eight weeks between PCV13 and PPSV23. For healthy 65-year-old or older adults, the CDC recommends a minimum interval of one year. However, this can be shortened to a minimum interval of 8 weeks, as is the case in transplant patients with immunodeficiency presented here [21]. In our study, we have chosen a vaccination interval of 6 months, as indicated by the current vaccination recommendation against pneumococci for risk groups of the Robert Koch-Institute in Germany [22].

A common problem, that arises in studies with pneumococcal vaccines, is the lack of a robust threshold to define effectiveness in adults, especially in the case of immunocompromised individuals. The WHO ELISA has been used intensely in evaluation and licensure of pneumococcal vaccines, especially pneumococcal conjugate vaccines in children. Based on ELISA results from three clinical studies on the seven-valent pneumococcal conjugate vaccine (Prevenar), the WHO defined a correlate of protection of 0.35 μg/mL for serotype-specific antibody concentrations for the license of vaccines against invasive pneumococcal disease in children [23]. But this threshold does not aim to imply protective status in an individual, nor could it be used to assume protection against other pneumococcal infections like pneumonia or otitis media, which may require higher antibody levels [24]. Other authors suggested the aforementioned cut-off should be higher [8, 25, 26]. In our study, we observed that for some serotypes, e.g. serotype 14, the baseline antibody concentrations were already much higher than 0.35 μg/mL.

Therefore, we tend to focus more on the relative increase of antibody levels after vaccination in comparison to baseline values as it was also done in a phase III clinical trial to evaluate the safety and immunogenicity of PPSV23 [15] and in a previous study in KTR investigating the immunogenicity of a sequential vaccination with PCV7 and PPSV23 [26]. The vaccination regiment in the current study comprises two different vaccine types, one polysaccharide vaccine (PPSV23) and one conjugate vaccine (PCV13). Polysaccharide antigens are large molecules consisting of repetitive epitopes. These molecules are not processed by antigen-presenting cells and interact directly with B cells, inducing antibody responses in the absence of T cells. However, T cell-independent responses have several limitations, including poor induction of immunological memory. In contrast, antibody responses against protein antigens are T cell-dependent and result in long-lived immunity due to the generation of immunological memory. Pneumococcal polysaccharide vaccines elicit responses that mainly induce IgG2 in adults, whereas both IgG1 and IgG2 responses are induced by pneumococcal conjugate vaccines [27]. In our study, we could demonstrate that all measured serotype-specific geometric mean antibody concentrations showed more than a twofold increase at month 12 compared to baseline, indicating a sufficient immunogenicity. This includes serotypes, that are an ingredient of only PPSV13 (6A), only PPSV23 (2, 9N, 11A) or both (3, 14). The time courses of serotype 3 and serotype 14 exemplify that the vaccination with PPSV23 hardly boosts the antibody concentrations. Whereas PCV13 doubles them, PPSV23 only slightly increases them. We observed the strongest fold-increase for serotype 9N, which is in line with the mentioned phase III study for PPSV23. The highest absolute antibody concentrations were recorded for serotype 14 as also described in previous studies on different vaccination strategies [8, 26].

Our global anti-pneumococcal antibody data support the courses of serotype-specific antibody concentrations. With exception of IgM (1.7-fold increase), all other antibody subclasses showed more than a twofold increase of GMC at month 12 compared to baseline. We compared our data to the results of a previous study of our group, which comprised a comparable cohort of 47 KTR, who received a single dose of PCV13 [11]. The determination of global antibody concentrations was done with same commercial ELISA in the same lab. The median age of the current cohort was slightly higher (57 years vs. 53 years) whereas the median interval between first vaccination and (last) kidney transplantation was a bit lower (38 months vs. 49 months). Immunosuppressive medication did not deviate significantly as the vast majority in both cohorts received triple therapy with tacrolimus, MPA and corticosteroids. The comparison revealed higher relative increases and absolute values for concentrations of all antibody types at month 12 indicating an enhanced and longer lasting serological immune response after sequential vaccination compared to a single vaccination with PCV13. Our previous study showed that serotype specific IgG antibody concentrations as determined by ELISA correlated significantly with their functional activity measured by the opsonophagocytic assay, indicating that serotype specific IgG antibodies are functional in this immunocompromised cohort [11]. However, it should also be noted that functional antibody concentrations may be lower than the concentration of binding antibodies presented in the current study.

There are only limited data on global and serotype-specific anti-pneumococcal antibody concentrations in healthy adults before and after vaccination and validated protective cut-off values are lacking [28]. Therefore, we compared our data with two groups of healthy adults [18, 19, 29]. The first cohort consists of vaccine naïve healthy individuals [17, 30]. The comparison revealed reduced global antibody concentrations (all subclasses) in KTR before vaccination. One month after first vaccination, comparable antibody concentrations to healthy adults were reached except for IgM. This finding was in line with our previous study on a single administration of PCV 13 in KTR [16]. But 12 months after vaccination, the GMC of all immunoglobulin classes matched or even exceeded those of vaccine naïve healthy adults. This did not apply to the same extent to our previous study [11]. To evaluate serotype-specific antibody concentrations, we compared our patient cohort with healthy adults, that were vaccinated with a single dose of PCV13 [19]. With respect to the tested serotypes, we could only compare antibody concentrations of serotypes 3, 6A and 14. Antibody concentrations were higher for all serotypes in healthy individuals at one month after administration of PCV13. However, at 12 months after vaccination with PCV13 antibody concentrations of serotypes that are also part of PPSV23 were comparable (serotype 3) or even higher (serotype 14) in kidney transplant recipients. In contrast, antibody concentrations of serotype 6A (only part of PCV13) were still much higher in healthy individuals.

Correlation analysis for all antibody subclasses revealed that values before vaccination were predictive of antibody concentrations after vaccination with highest correlation coefficients for IgM antibodies. Overall, the strongest correlation was seen between IgG and IgG2 antibody concentrations, reaching highly significant results (p < 0.001) at each timepoint. *Robbins *et al*.* investigated the immunogenicity of PCV13 in an adult cohort with common variable immunodeficiency (CVID) or IgG subclass deficiency [31]. They observed that higher global IgG and IgG2 values at baseline were associated with protection at one year after vaccination. These findings are in line with a study on patients with systemic lupus erythematodes, for whom higher global IgG2 serum concentrations were associated with long-lasting protection three years after sequential PCV13/PPSV23 vaccination [32]. In general, IgG2 is known to play a key role in the defense against pneumococcal infections [33].

Decreased anti-pneumococcal IgA and IgM levels have been observed in healthy adult blood donors [18], but have also been associated with a pronounced rate of respiratory infections in patients with CVID [34] and primary antibody deficiency (PAD) [35]. It is also known that individuals characterized as having an intact humoral response based on measurement of serotype-specific IgG concentration can still display impaired anti-pneumococcal IgM and IgA levels [36]. Thus, an additional determination of anti-pneumococcal IgM and IgA concentrations could yield more precise information on the humoral response to pneumococcal vaccines in individuals, but the clinical benefit is questionable [36].

The global anti-pneumococcal IgG ELISA shows a good correlation to the GMC of serotype-specific antibodies, with exception of serotype 2, for most time-points in our study and was also reported previously [11]. This commercial assay therefore serves as a cost-effective and easy tool to monitor the humoral immune response to pneumococcal vaccination in clinical routine [28]. But to get more insight into serotype-specific serological responses, especially in the case of low-level global anti-pneumococcal IgG concentrations, serotype-specific WHO ELISA is required [28]. In particular, serotype 3 remains a dominant cause of invasive pneumococcal disease (IPD) in Europe [37]. In our study, only 56.5% of recipients showed a twofold increase of antibodies against serotype 3 after 12 months, compared to baseline. This is approximately 10% less than the mean value for the other serotypes. Moreover, concentrations were by far the lowest of the tested serotypes. Since serotype 3 is a main driver of IPD in Germany, these results corroborate the well-known lack of vaccine-protection [37–39]. However, for a more accurate assessment of this problem, a longer follow-up of recipients and larger group of participants are necessary.

We compared the vaccine-induced immune responses with clinical patient data. We observed that patients with MPA treatment had significantly lower global IgG and IgG2 antibody concentrations compared to patients without MPA intake. We also tested, if we could confirm this finding for serotype-specific antibodies, but although we did see the same trend, it did not reach significance (data not shown). This may be explained by the small size of both groups (*n* = 33 vs. *n* = 13) in the sense that statistical significance was only reached by a summed effect of global antibody concentrations. Interval between (last) kidney transplantation and first vaccination were the only other variable with a significant association with global antibody GMC. In that case we recorded a positive correlation meaning that a longer interval, which normally implicates reduced immunosuppressive treatment, was associated with higher IgG and IgG2 GMCs. However, regression analysis revealed that only MPA treatment proved to be significant for IgG and IgG2 GMC at month 7 and month 12 after first vaccination, highlighting its relevance in case of long-term vaccine immunogenicity. This is in line with previous studies, which generally describe the dominant effect of MPA treatment on humoral immune responses [40] or specify its impact on serological responses after pneumococcal vaccination [11]. Mycophenolic acid inhibits the generation of guanine nucleotides. Unlike different other cell types (e.g., neurons, hepatocytes and renal cells), lymphocytes can only generate guanine nucleotides de novo. Therefore, they are a rather specific target of MPA, which leads to reversible inhibition of B and T cell proliferation. This explains the negative effect on humoral immune responses as described previously and in the current study. The calcineurin inhibitor tacrolimus primarily affects T cell functionality but can indirectly inhibit B cell functions that depend on CD4^+^ T cell interaction. Accordingly, tacrolimus is more likely to impair the efficacy of T cell-dependent conjugate vaccines like PCV13 than T cell independent polysaccharide vaccines. Glucocorticoids, which are also part of the conventional immunosuppressive triple therapy in kidney transplantation, have pro-apoptotic effects on B and plasma cells. The combination of MPA, tacrolimus and glucocorticoids therefore leads to a substantial suppression of humoral immunity [40, 41]. However, our data shows that MPA has the strongest influence on the production of anti-pneumococcal antibodies.

In this subgroup of organ transplant recipients, a third vaccination may be required to reach equivalent antibody concentrations. Pneumococcal booster vaccination has already been shown to improve overall immune protection against pneumococcal disease in immunocompromised patients such as HIV-positive adults and is highly recommended for adults with chronic obstructive pulmonary disease [42, 43]. Further studies are necessary to determine the most suitable vaccine for booster vaccination in kidney transplant recipients and to compare its impact on the incidence of pneumococcal disease.

There are several limitations to the study that should be acknowledged. First, the study lacked a control group that received the same vaccine sequence, which would have allowed a more robust comparison of the effectiveness and safety of the PCV13 and PPSV23 sequential vaccination. Direct comparison of PPSV23 vaccine efficacy between KTR and the healthy blood donor population is not ideal. Second, the follow-up period of 18 months may not be sufficient to assess the long-term effectiveness. Vaccine efficacy may decrease after 5 years, so CDC recommends booster vaccination after 5 years for adults 65 years and older, who received PCV13 at any age and PPSV23 before age 65 [21]. Third, the study was conducted in a single center in Germany, which may limit the generalizability of the findings to other regions or populations with different characteristics, prevalent serotypes or risk factors. However, we found that all measured serotype-specific geometric mean antibody concentrations showed more than a twofold increase at month 12 compared to baseline, indicating sufficient immunogenicity of sequential vaccination in KTR. In addition, diminished antibody rise was narrowed down to therapy with MPA.

To conclude, sequential vaccination with PCV13 and PPSV23 in kidney transplant recipients results in a superior antibody response compared with single vaccination. MPA treatment significantly reduced the antibody response in contrast to any other immunosuppressive therapy.

## Data Availability

The data presented in this study are available on request from the corresponding author. The data are not publicly available due to privacy restrictions.
